# Supercapacitor based on polymeric binary composite of polythiophene and single-walled carbon nanotubes

**DOI:** 10.1038/s41598-022-15477-z

**Published:** 2022-07-04

**Authors:** Azza Shokry, Marwa Karim, Marwa Khalil, Shaker Ebrahim, Jehan El Nady

**Affiliations:** 1 Department of Materials Science, Institute of Graduate Studies and Research, Alexandria University, 163 Horrya Avenue, El-Shatby, P.O.Box 832, Alexandria, Egypt; 2 Physics Department, Faculty of Science, Alexandria University, Moharram Bek, P.O. Box 21511, Alexandria, Egypt; 3 Department of Nanotechnology and Composite Materials, Institute of New Materials and Advanced Technology, City of Scientific Research and Technological Applications (SRTA-City), New Borg El Arab City, P.O. Box 21934, Alexandria, Egypt; 4 Electronic Materials Department, Advanced Technology and New Materials Research Institute, City of Scientific Research and Technological Applications (SRTA-City), New Borg El-Arab City, P.O. Box 21934, Alexandria, Egypt

**Keywords:** Energy science and technology, Materials science

## Abstract

The aim of this work is to fabricate supercapacitor electrode based on poly (3-hexyl-thiophene-2, 5-diyl) (P3HT) and single-walled carbon nanotubes (SWCNTs) nanocomposites with different ratios onto a graphite sheet as a substrate with a wide voltage window in nonaqueous electrolyte. Structural, morphological and electrochemical properties of the prepared nanocomposites of P3HT/SWCNTs were studied and discussed. The electrochemical properties included cyclic voltammetry (CV), galvanostatic charging-discharging (GCD), and electrochemical impedance spectroscopy (EIS) were investigated. The obtained results indicated that P3HT/SWCNTs nanocomposite possesses higher specific capacitance than that present in its individual component. The high electrochemical performance of the nanocomposite was due to formation of microporous structure which facilitates ions diffusion and electrolyte penetration in these pores. The morphological micrographs of the purified SWCNTs had buckypaper structure while the photomicrographs of P3HT/SWCNTs showed that SWCNTs appear behind and front of the P3HT nanospheres. The specific capacitance of 50% SWCNTs at 0.5 Ag^−1^ was found to be 245.8 Fg^−1^ compared with that of pure P3HT of 160.5 Fg^−1^.

## Introduction

Since the discovery of the conducting polymers like poly (3‐hexylthiophene) (P3HT), polypyrrole and polyaniline, many scientists have been working on finding applications for these polymers as light emitting diodes^1,2^, adsorbents^3,4^, electrochromic devices^5^, sensors^6^, and supercapacitors^7,8^. Electrochemical supercapacitors as promising energy storage devices provide low energy density, high power density, fast charging discharging rate and long cycle lifetime^9,10^. Supercapacitors (SC) or ultracapacitors have pointed out to capacitors with high-surface area of the electrodes. SCs can harvest energy in very short time to provide spurt of energy when a quick charge is required. Based on charge and discharge mechanism, supercapacitors are categorized into electric double-layer supercapacitors (EDLCs), pseudosupercapacitors (PSC) and hybrid supercapacitors. EDLCs are also called electrostatic capacitors and the charge storage in EDLCs takes place at the electrode/electrolyte interface through the electrostatic charge adsorption mechanism^11,12^. The specific capacitance of this type relies on the specific surface area, pore size, pore shape, morphology and electrical conductivity. In PSCs store charges via fast and reversible redox or Faradic reactions occurring on metal oxides or conducting polymers. The reversible redox reactions occurred at the surface of the electrode materials produce high energy density compared to EDLCs^10,13,14^.

Among PSC materials, conductive polymers and transition metal oxides are promising materials as SC electrodes. P3HT, polypyrrole and polyaniline are interested in the field of energy storage due to their electrochemical reversibility, doping–dedoping during the charge–discharge process, and high electrical conductivity^9,15^. P3HT as a soluble conducting polymer is suitable and appropriate for the fabrication of supercapacitor electrode because of its pseudosupercapacitance behavior, unique electrical conductivity and high energy density^16^. In addition, P3HT combined with carbon nanostructures can store the charge at electrical double layer formed at electrode/electrolyte interface. However, swelling and shrinkage of the P3HT in the electrolytes lead to mechanical degradation^17–19^.

Single-wall carbon nanotubes (SWCNTs) and multi-walled carbon nanotubes (MWCNTs) have used as electrodes for the supercapacitors due to their unique hollow structure, electronic conductivity, thermal stability and mechanical strength^20,21^. Many efforts have been made to fabricate P3HT/SWCNTs electrode because of their high specific surface area, which can expose entirely either basal graphite planes or edge planes to the electrolyte^22–25^. Dhibar et al. prepared graphene/SWCNTs/poly(3-methylthiophene) ternary nanocomposite supercapacitor electrodes and achieved a specific capacitance of 551 F/g with a small voltage window between 0 and 0.8 V^23^. Zhou et al. grafted and fabricated poly(3-oligo(ethylene oxide)) thiophene onto SWCNTs supercapacitor electrode in the negative window from − 0.9 to − 0.1 V and obtained a specific capacitance of 399 F/g^25^.

Herein, we report specific capacitances of 245 Fg^−1^ at 0.5 Ag^−1^ for the fabricated supercapacitor electrode with a wide voltage window based on P3HT/SWCNTs nanocomposite electrode onto a graphite sheet as a substrate in 0.1 M LiClO_4_. P3HT/SWCNTs nanocomposites with different ratios by physical blend were prepared. The electrochemical properties of these electrodes were investigated via CV, GCD, and EIS measurements. The obtained results indicated that P3HT/SWCNTs nanocomposite electrodes possess higher specific capacitance than the pristine component. The good electrochemical performance of the nanocomposite is attributed to π–π interactions between SWCNTs and P3HT and formation of microporous structure to facilitate rapid ions diffusion and electrolyte penetration in these pores.

## Results and discussion

### Structural property

**Figure 1:**
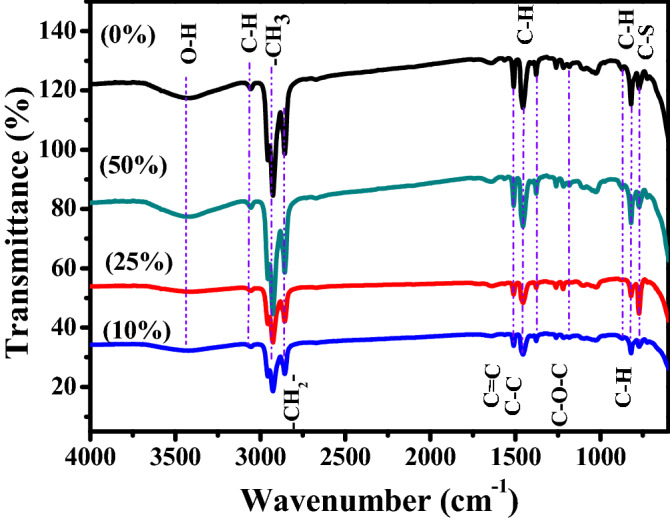
FTIR spectra of P3HT, and P3HT/50% SWCNTs, P3HT/25% SWCNTs and P3HT/10% SWCNTs nanocomposites.

In order to investigate the changes in the chemical structures of P3HT, SWCNTs and P3HT/SWCNTs nanocomposites with different SWCNTs ratios, infrared and Raman spectra are analyzed as described as follow. Figure 1 illustrates the absorption peaks of the infrared spectra of P3HT and P3HT/10% SWCNTs, P3HT/25% SWCNTs and P3HT/50% SWCNTs nanocomposites. The absorption peak at 3448 cm^−1^ belongs to the O–H stretching vibration. The small peak at ~ 3051 cm^−1^ is ascribed to the C–H aromatic stretching vibration of the thienyl ring. The features in 2922–2855 cm^−1^ range correspond to the –CH_3_ and –CH_2_– stretching vibrations of the hexyl side chains^26–28^. Characteristic band at 1509 cm^−1^ is corresponded to C = C vibration of quinoid unit of the polymer chain. In addition, the band at 1452 cm^−1^ is ascribed to the C–C ring stretching. Moreover, C–H and C–O–C stretching vibrations appear at 1310 and 1175 cm^−1^, respectively. The weak bands at 878 and 825 cm^−1^ are assigned to the C–H out-of-plane stretching and bending vibrations of the thiophene ring, respectively. The small band at 670 cm^−1^ is due to C–S bending of P3HT. FTIR spectra of P3HT/SWCNTs nanocomposites with different ratios of SWCNTs have the same features of pristine P3HT without shifting. This indicates that the P3HT/SWCNTs nanocomposites are formed because of the simple π–π stacking interactions instead of other stronger interactions between P3HT and SWCNTs^29^.

**Figure 2:**
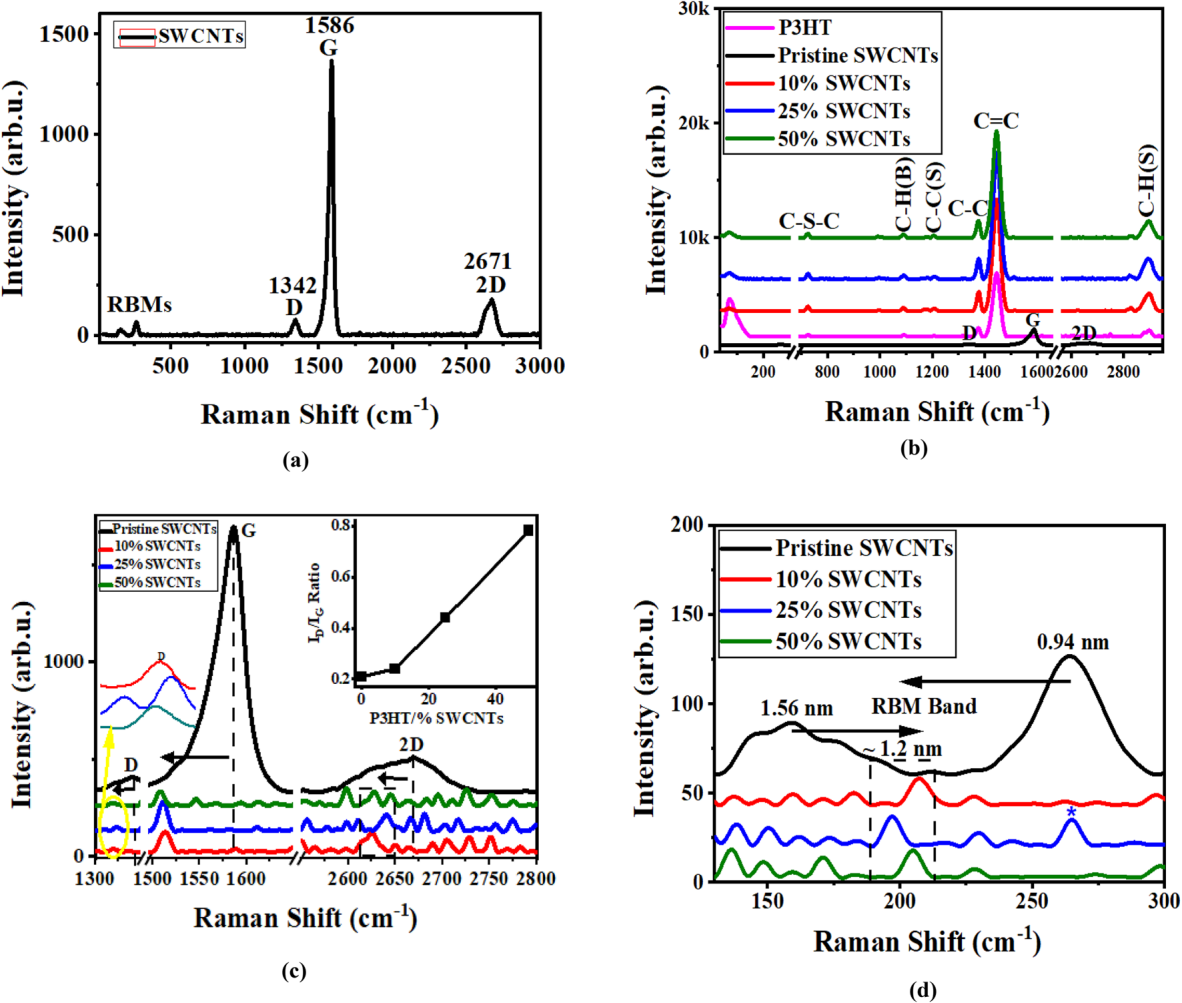
Raman spectra of (**a**) pristine SWCNTs, (**b**) P3HT/SWCNTs nanocomposites with different ratios of SWCNTs, (**c**) analysis of the D, G and 2D band, Inset of (**c**) presents the I_D_/I_G_ ratios and (**d**) analysis of the RBMs band.

Raman spectroscopy is conducted to investigate the structural configurations of the carbon-based materials. Figure 2 displays the Raman bands of SWCNTs, P3HT, and P3HT/SWCNTs nanocomposites with different SWCNTs ratios. As illustrated in Fig. 2a, the pristine SWCNTs shows that Raman spectrum exhibits the radial breathing modes (RBMs) with two peaks at 264 and 158.8 cm^−1^, a sharp order G band at 1586 cm^−1^, a weak disorder D band at 1342 cm^−1^, and a small 2D band at 2671 cm^−1^^25^. Raman spectra of the pure P3HT and P3HT/SWCNTs nanocomposites with different ratios are depicted in Fig. 2b and compared with the pristine SWCNTs bands. Firstly, the pure P3HT shows main dominant peaks at around 1444 and 1375 cm^−1^ corresponding to the characteristic C=C and C–C in-phase vibrations of the thiophene rings, respectively^25^. There are also two small bands observed at 2895 and 1091 cm^−1^ assigned to the C–H stretching and bending, respectively. In addition, a small peak at 1207 cm^−1^ is attributed to C–C stretching and the band at 725 cm^−1^ is corresponded to C–S–C ring deformation^30^.

Notably, the P3HT/SWCNTs nanocomposites spectra exhibit the characteristic peaks of the pure P3HT as well as the G, D and 2D bands of SWCNTs, respectively. Figure 2c presents the D, G, and 2D bands in the Raman spectra of the pristine SWCNTs and PHT/SWCNTs nanocomposites. The sharp order G band is associated with the sp^2^ hybridized carbon atoms in the nanotubes wall and the weak disorder D band is sensitive to the sidewall sp^3^ hybridized carbon atoms created by covalent functionalization (i.e., indicates the existence of structural defects on the surfaces of SWCNTs)^24^. The resonant 2D band is considered as an overtone of the D band. The surface modifications and the functionalization degree on the nanotube walls can be evaluated via the I_D_/I_G_ ratio. The high intensity values of this ratio enhance the surface functionalization on the nanotube walls^31,32^. As shown in the inset of Fig. 2c, the I_D_/I_G_ ratios are calculated for both the pristine SWCNTs and the P3HT/SWCNTs nanocomposites with different SWCNTs ratios. For P3HT/SWCNTs nanocomposites, I_D_/I_G_ ratio is increased as ratio of the SWCNTs increases and P3HT is anchored on the surface of the SWCNTs. It is noted that 50% SWCNTs nanocomposite exhibits the highest homogenous mixing between the P3HT and SWCNTs. G band position can be used to evaluate the charge transfer in SWCNTs composites^33–35^. The redshift is a result of the charge transfer from the electron donors in P3HT to the π-system of the SWCNTs^33–35^. Thus, the redshift of the G band in the P3HT/SWCNTs nanocomposites is due to π-π stacking interaction between P3HT and SWCNTs. Hence, it is clearly visible that the 50% SWCNTs nanocomposite has the highest redshift value and thus has the better charge transfer interaction and facilitates the ions diffusion of the electrolyte.

The analysis of the RBMs of the pristine SWCNTs and P3HT/SWCNTs nanocomposites with different SWCNTs ratios is depicted in Fig. 2d. The RBMs are corresponded to the coherent vibration of the carbon atoms in the radial direction of the nanotubes (i.e., as if the nanotubes are “breathing”)^35^. The RBMs of SWCNTs are used to estimate their diameters (d) via the positions of the Raman peaks (ω_RBM_) using c/ω_RBM_^36^ (where the constant c is 248.3 nm/cm^−1^^37^. It is observed that there are two diameters for the pristine SWCNTs with 0.94 and 1.56 nm. On the other hand, the P3HT/SWCNTs nanocomposites exhibit RBMs with only one peak, which is shifted to different peak position. The presence of the RBMs in the nanocomposites spectra confirms the embedded of the SWCNTs in the composite. The appearance of this peak in the nanocomposite leads to the translation of the SWCNTs to a dominate mean diameter^38^. Hence, the estimated mean diameters for the 10, 25, and 50% SWCNTs nanocomposites are 1.2, 1.26, 1.21 nm, respectively. It is noticed that the small-diameter SWCNTs in the composite are apparently eradicated expected for the 25% SWCNTs sample and the presence of the peak with the blue asterisk is attributed to the unreacted SWCNTs in the composite.

### Morphological property

**Figure 3:**
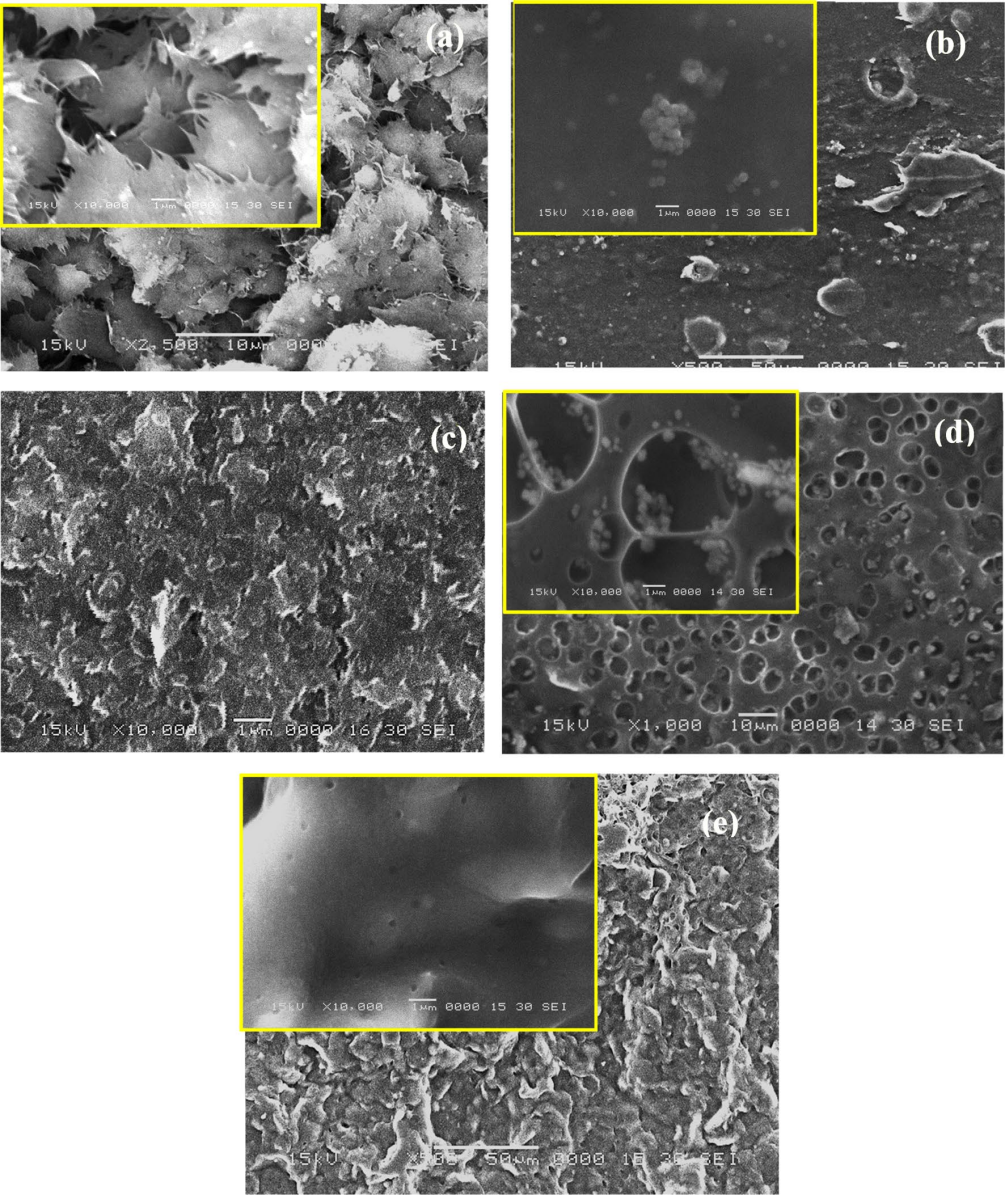
SEM images of: (**a**) purified SWCNTs, (**b**) pure P3HT, (**c**) P3HT/10% SWCNTs, (**d**) P3HT/25% SWCNTs and (**e**) P3HT/50% SWCNTs. The inset images are the samples at high magnification.

Figure 3 illustrates the scanning electron micrographs of SWCNTs, pure P3HT and P3HT/SWCNTs nanocomposites at different SWCNTs contents. The SEM micrographs of the purified SWCNTs sample at different magnifications shown in Fig. 3a have a paper-like morphology or buckypaper structure. This structure is the product of the filtration step during the purification process of SWCNTs synthesis. After the filtration step, SWCNTs are closer to each other to form rigid bundles. These bundles are hard to see by SEM at high magnifications. However, as shown in Fig. 3a some bundles are observed at the boundaries, which serve as a connection between the sheets of the buckypaper^39^.

The pure P3HT shows randomly and dispersed microgranulars and small particles structure as presented in Fig. 3b. The size of microgranulars is about 0.5 μm. The SEM images in Fig. 3c–e illustrate the photomicrographs of P3HT/10% SWCNTs, P3HT/25% SWCNTs and P3HT/50% SWCNTs, respectively. P3HT/10% SWCNTs nanocomposite image displays a compact and homogenous layer due to the dominant component of P3HT as shown in Fig. 3c. On the other hand, at high percentages of SWCNTs of 25 and 50% there are different phases in the formed nanocomposites. There are large numbers of voids and polythiophene particles or granules shielded and masked the SWCNTs as displayed in Fig. 3d. Generally, there are tubular and some globular structures for P3HT/SWCNTs nanocomposites. As the percentage of SWCNTs in P3HT/SWCNTs increases, the tubular structure is observed indicating that SWCNTs act as hard templates onto P3TH deposited to form the tubular morphology^40^.

**Figure 4:**
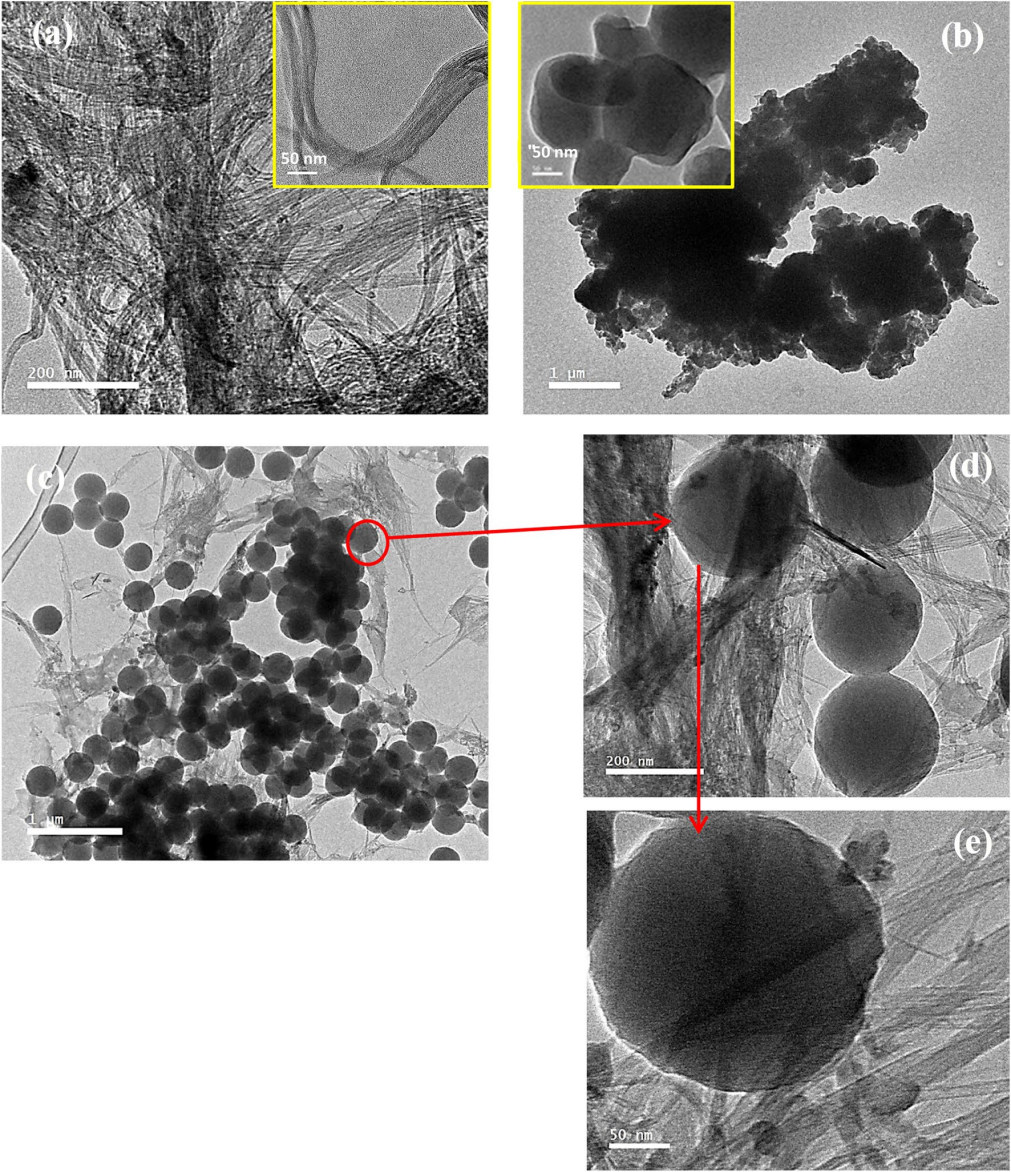
TEM images of: (**a**) purified SWCNTs, (**b**) pure P3HT, the inset image is the sample at higher magnification. (**c–e**) P3HT/50% SWCNTs at deferent magnifications.

HR-TEM is carried out to investigate the interior features of purified SWCNTs, pure P3HT and P3HT/50% SWCNTs nanocomposite as shown in Fig. 4. Figure 4a illustrates TEM image of SWCNTs with a large quantity of bundles with a diameter less than 25 nm (inset of Fig. 4a). The bundles of these nanotubes are relative clean, smooth surface and a coil-like structure. Figure 4b presents TEM image of pristine P3HT with typical nanosheets or spherical structure. These sheets are coagulated and aggregated together with small particles and granules. The P3HT/50% SWCNTs nanocomposite images have a phase separation of their two components (nanotubes and nanosheets nanospheres) and in some regions the nanotubes appear behind and front of the P3HT spheres as illustrated in HR-TEM images shown in Fig. 4c–e.

### Electrochemical performance of P3HT/SWCNTs composite

**Figure 5:**
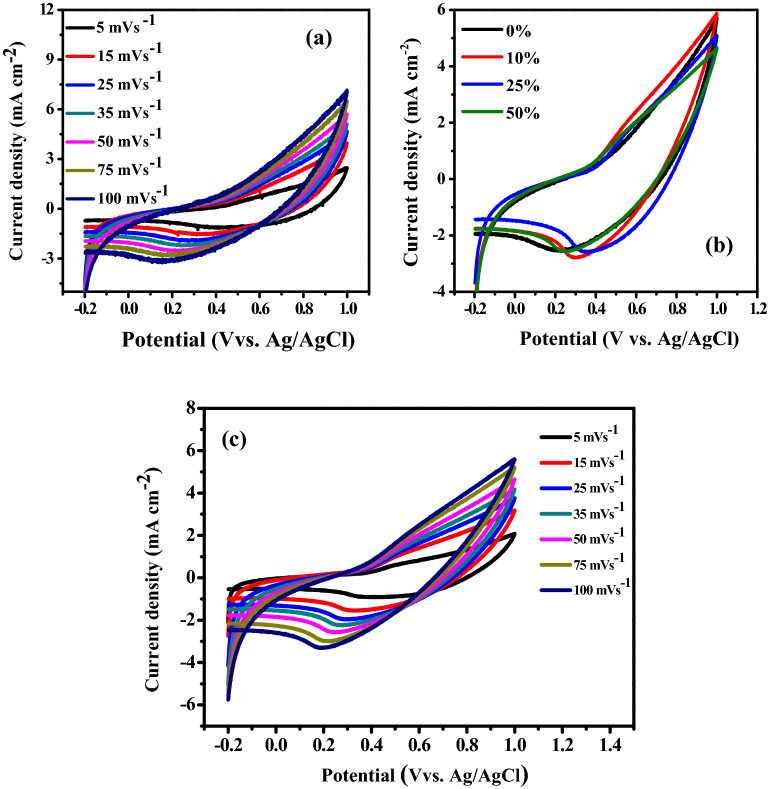
(**a**) CV curves of P3HT at different scan rates, (**b**) CV curves of P3HT/SWCNTs electrodes with different SWCNTs ratios at 50 mV s^−1^ and (**c**) CV curves of P3HT/50% SWCNTs at different scan rates**.**

The electrochemical performance of the fabricated supercapacitor electrodes of the pristine P3HT and P3HT/SWCNTs nanocomposites with different ratios are evaluated by CV, GCD and EIS using three-electrode configuration. The CVs (third cycle) are carried out in the potential range from − 0.2 to 1 V vs. Ag/AgCl with different scan rates from 5 to 100 mVs^−1^ in 0.1 M LiClO_4_ in acetonitrile as shown in Fig. 5a. The fabricated electrode has a stable shape of CV curves. There is a single oxidation peak located at 1.0 V and one reduction peak at around 0.6 V at scan rate 5 mV/s. This indicates of the Faradic pseudocapacitive nature of the electrode^41^. However, the voltammograms obtained do not show clearly the anodic peaks and a small reduction peak attributable to the reduction of the film deposited on the electrode. This can be explained based on the participation of capacitive current. The current intensity of the anodic and cathodic peak increases with scan rate. The difference between the oxidation potential and the reduction increases with the scan rate. The proportionality of the peak intensity to the scan rates suggests that the oxidation of electroactive P3HT on the electrode surface is limited by a diffusional process^42^. For the CV curves of pristine P3HT shown in Fig. 5a, the current increases with scan rate and maintains the shape of CV curves, suggesting that there is a good rate of the capability^43^.

It is noted that the values of area of the CVs of P3HT/SWCNTs nanocomposites are larger than that of pristine P3HT as shown in Fig. 5b. In addition, there is a slightly effect on SWCNTs addition on the CV behavior due to the nature of electric double layer of the carbon materials. It is observed that the electrochemical supercapacitors containing P3HT component have suffered from relatively large internal resistance^44^. Figure 5c depicts the CV curves of P3HT/50% SWCNTs nanocomposite at different scan rates. At low scan rates, the ions from the electrolyte can easily diffuse in the microporous surface and bind to the active sites. On the other hand, some active pores on the surface becomes impenetrable for electrolyte ions and charge storage, which describes a decrease in the specific capacitance at high scan rates. Moreover, for this nanocomposite electrode there is a slightly shift in the redox peaks at high scan rates, indicating irreversibility of the electrode materials^14,45^.

**Figure 6:**
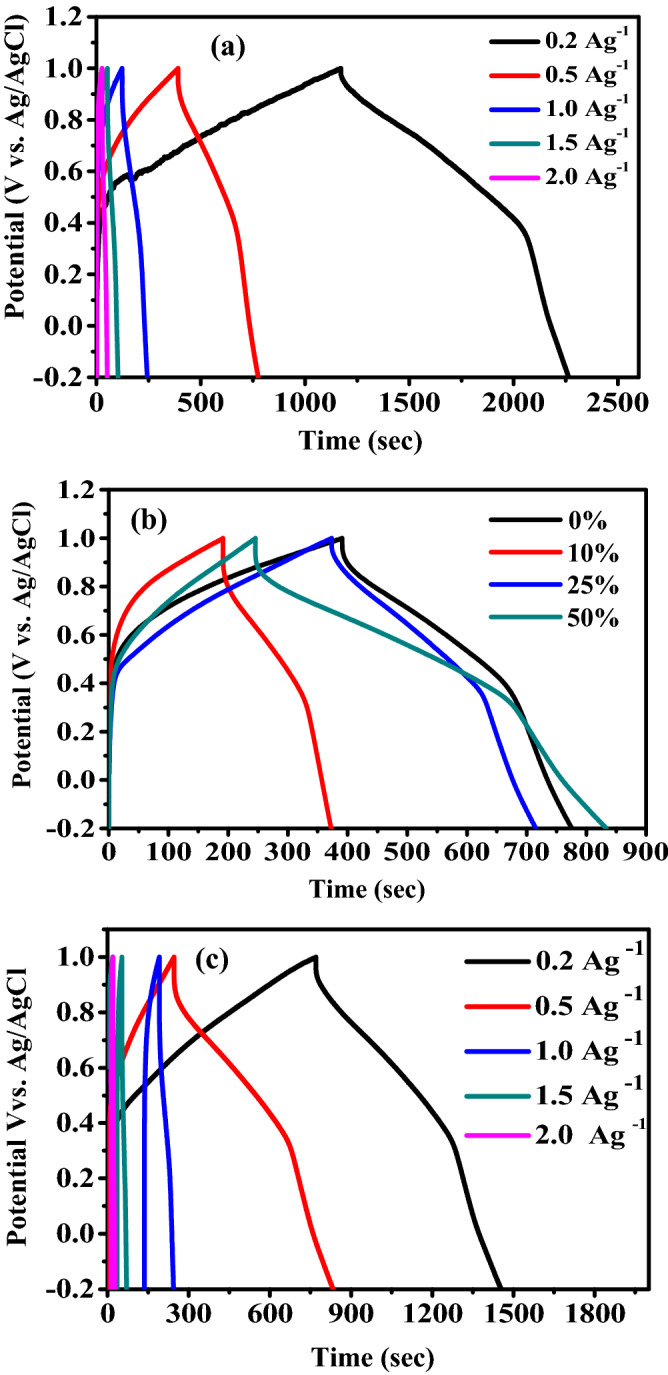
(**a**) GCD curves of P3HT at different current densities, (**b**) GCD curves of P3HT/SWCNTs electrodes with different SWCNTs ratios at 0.5 Ag^−1^ and (**c**) GCD curves of P3HT/50% SWCNTs at different current densities.

The GCD curves of pure P3HT and P3HT/SWCNTs electrodes at current densities of 0.2, 0.5, 1.0, 1.5 and 2.0 Ag^−1^ in the potential window range from − 0.2 to 1.0 V vs. Ag/AgCl are illustrated in Fig. 6. In addition, the effect of SWCNTs content in P3HT/SWCNTs electrodes at 0.5 Ag^−1^ on GCD is displayed and investigated. The curves exhibit semisymmetric triangular shapes and the potential-time relationships are deviated from the linearity, indicating pseudocapacitive contribution and the capacitance of P3HT/SWCNTs originates from pseudocapacitance plus electric double layer capacitance of SWCNTs^46^. There are two different regions appeared in the discharge curves. Fast potential drop is initially occurred and followed by a slow potential decay. The fast decay is attributed to the internal resistance of the electrode and the latter represents the capacitive feature of the pseudocapacitive electrode^46^. The nonlinear curves of P3HT/50% SWCNTs electrode at different current densities have pseudocapacitance behavior. Compared with others P3HT/SWCNTs electrodes, P3HT/50% SWCNTs electrode has lower internal resistance and higher pseudocapacitance. Hence the discharge times of P3HT/10% SWCNTs, P3HT/25% SWCNTs and P3HT/50% SWCNTs are 180.45, 343.45 and 589.82 s, respectively. In addition, the GCD curves are not perfectly symmetrically attributed to the electrochemical reversibility of the P3HT/SWCNTs nanocomposite similar to battery-like materials^47^.

The specific capacitance is calculated from the GCD curves. The P3HT electrodes show that the values of capacitance are declined with increase of the current density (Fig. 6a). From the charge–discharge curves, it is observed that there is a significant effect for SWCNTs ratio in P3HT/SWCNTs nanocomposites on performance of the supercapacitor electrodes as illustrated in Fig. 6b. The electrode based on 50% SWCNTs has a longer discharging time than other ratios in P3HT/SWCNTs nanocomposites. The specific capacitance of P3HT/50% SWCNTs electrode at 0.5 A g^−1^ is found to be 245.8 Fg^−1^ while the specific capacitance of pure P3HT electrode is 160.5 Fg^−1^ (Fig. 6c). These results could be ascribed to the rapid insertion/extraction of electrolyte ions and the voids and pores morphology of the 50% SWCNTs electrode surface. This leads to an enhancement in the effective surface area and the conductivity^48^. On the other hand, P3HT/10%SWCNTs electrode has a minimum specific capacitance of 75.5 Fg^−1^ where this electrode has a compact and dense surface as dedicated in the SEM images.

**Figure 7:**
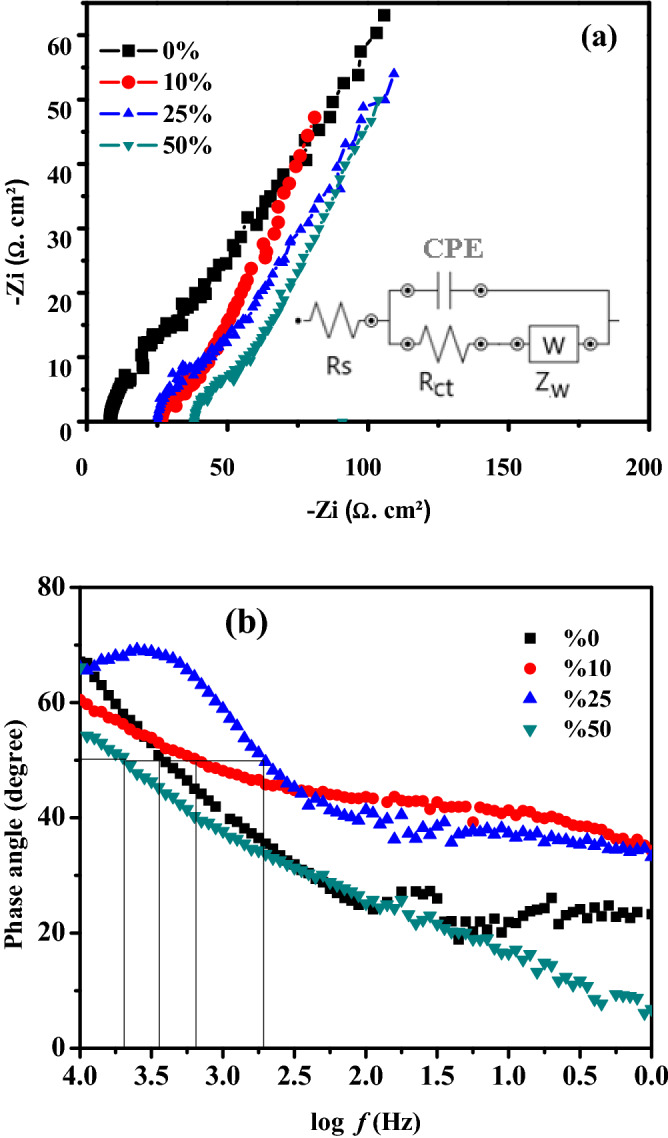
Nyquist (**a**) and Bode phase angle (**b**) plots of P3HT/SWCNTs electrodes with different SWCNTs ratios.

The charge transport and ions diffusion through the fabricated P3HT/SWCNTs electrodes based can be investigated and studied using EIS measurement. Figure 7 illustrates the Nyquist and Bode phase plots of P3HT/SWCNTs electrodes with different SWCNTs ratios in frequency range from 10 kHz to 0.01 Hz. Nyquist plots are analyzed based on the equivalent circuit achieved with Nova software as noted in the inset of Fig. 7a. R_s_ represents the total resistance of electrolyte and electrode^49^. The constant phase element (CPE) is pseudocapacitor component, R_ct_ corresponds to the charge transfer resistance and Zw is the Warburg impedance which is attributed to the ions diffusion and electrolyte penetration^50–52^. Nyquist plots exhibit a semi-circle in the high frequency region and a straight part in the low frequency region. This semicircle is attributed to charge transfer resistance at the electrode–electrolyte interface, and the straight line represents the diffusion mechanism of the electrolyte through the electrode surface. It is found that R_s_ values are 7.9, 27.1, 25.7, and 37.3 Ω for 0%, 10%, 25%, and 50% SWCNTs in P3HT/SWCNTs electrodes, respectively. R_ct_ values of P3HT and P3HT/50% SWCNTs are evaluated to be 17 and 8 Ω, respectively. P3HT/50% SWCNTs electrode at low frequency region displays larger slope than P3HT electrode. The microporous structure of P3HT/50% SWCNTs electrode offers a high surface area and can facilitate rapid ions diffusion and electrolyte penetration in these pores, which improves the specific capacitance^50^.

Bode phase angle plots are the second main format of EIS presentation. The ideal capacitor is expected to have phase angle of 90°. The phase angle of the highest specific capacitance sample (50% SWCNTs) is 55° and is lower than the other samples (Fig. 7b) suggesting a high ionic permeability occurs at low frequencies. Therefore, P3HT/50% SWCNTs electrode is permeable to ions and leads to an increase of the ionic resistance. The capacitor response frequency (*f*_0_) is characterized as the position of equal resistive and capacitive, and the relaxation time constant (τ_0_) is defined as the minimum time for discharging the energy from the supercapacitor. The value of *f*_*0*_ is obtained from the Bode phase angle plots at the position of 45° and τ_0_ is calculated using the formula of τ_0_ = 1/*f*_0_^10,53^. The relaxation time constant represents the transition from pure resistive to pure capacitive behavior for the electrochemical capacitor. Relaxation times of 0.3, 0.6, 1, and 0.2 s are obtained for the nanocomposite electrodes with 0%, 10%, 25%, and 50% SWCNTs, respectively. These values show that the SC electrodes can be fully discharged within a very short time with an efficiency of more than 50%. Low values of τ suggest that the fast redox reactions result in quick ions transfer between electrodes and electrolyte^10,54^.

**Figure 8:**
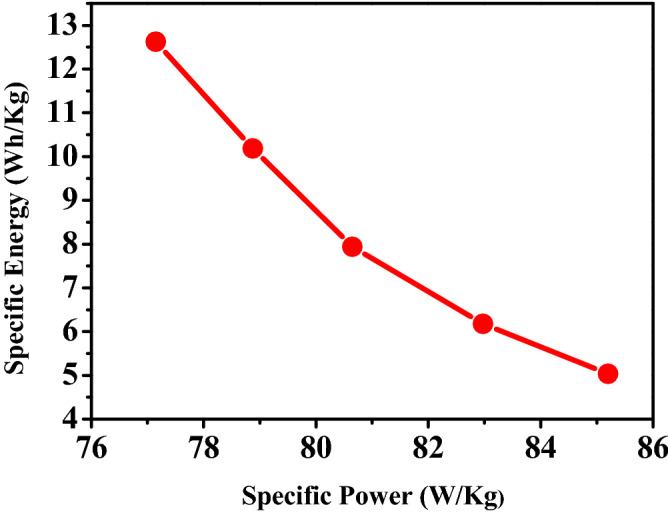
Ragone plot of P3HT /SWCNTs assembled nanocomposite supercapacitor electrode.

The Ragone plot of specific power density against specific energy density for P3HT/50% SWCNTs electrode is shown in Fig. 8. The obtained curve indicates that the specific power density is declined upon increasing the specific energy density. The energy density is attained about 50.8 Wh kg^−1^ at a power density of 308.7 W kg^−1^, which are remarkably high value. In order to represent an actual device, the energy density and power density for three electrodes system are divided by 4 to be equal to 12.7 Wh kg^−1^ and 77.2 W kg^−1^, respectively.

**Figure 9:**
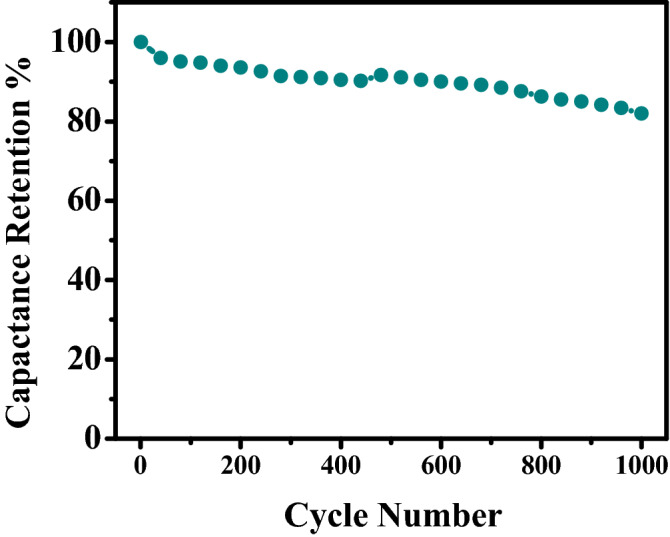
Cycling stability of the P3HT/50% SWCNT nanocomposite supercapacitor electrode at 1 A g^−1^ for 1000 GCD cycles.

The long-term stability of the P3HT/50% SWCNTs nanocomposite electrode is examined by GCD cycling at 1 A g^−1^ as presented in Fig. 9. P3HT/50% SWCNTs electrode keeps the capacitance retention of 80.5% after 1000 cycles. This result indicates that the P3HT/SWCNTs nanocomposite film has long-term cycle stability and could be used as an electrode material for supercapacitors. The stability of pure P3HT during charge–discharge cycles is poor and exhibits a fast decay of specific capacitance due to the degradation of P3HT which occurs mainly due to charge trapping and volume expansion and contraction during intercalation and deintercalation of electrolyte ions into the matrix of the polymer^50^. In addition, charge trapping is also reduced in the nanocomposites as SWCNTs can act as a current collector. The low stability of P3HT/SWCNTs nanocomposite electrode comes back to the irreversible redox and high Rs value.

The comparison between supercapacitor electrodes previously reported and P3HT/50%SWCNTs electrode are summarized in Table 1. We used a wider potential window and acetonitrile electrolyte than other PHT/SWCNTs electrodes which leads to higher energy density as shown in Table 1.

**Table 1:** Supercapacitor parameters of various polymers/SWCNTs electrodes presented in literatures and the present work.

Electrode materials	Electrolyte	Potential window (V)	Specific capacitance	Energy density (Wh kg^−1^)	Cycle life retention	Year/Ref.
PANI/SWCNTs	1 M H_2_SO_4_	− 0.2 to 0.8	446 F g^−1^ at 1 A g^−1^	19.45	98% (13,000)	2017/^55^
PANI /SWCNTs/cloth	1 M H_2_SO_4_	− 0.2 to 0.8	410 F g^−1^ at 0.5 A g^−1^	26.6	90% (3000)	2011/^56^
PPy/TiO_2_ /SWCNTs	1 M KCl	-0.3 to 0.3	282 F g^−1^	1.0	63.9% (1000)	2014/^57^
PANI /SWCNTs	1 M H_2_SO_4_	0.0 to 0.7	485 F g^−1^ at 5 mA cm^−2^	228	94% (1500)	2006/^58^
SWCNTs/SBS	Ionic liquid [EMIM][NTf2]	0.0 to 3.0	15.2 F cm^−3^ at 0.021 A cm^−3^	−	93% (1000)	2018/^59^
PEDOT-PSS/SWCNTs	1 M NaNO_3_	0.0 to 1.0	104 F g^−1^ at 0.2 A g^−1^	2.80	90% (1000)	2011/^23^
Gr/SWCNTs /PMT	1 M KCL	0.0 to 0.8	551 F g^−1^ at 0.5 A g^−1^	48.97	93% (1000)	2014/^23^
PD2ET/SWCNTs	1 M KCl	− 0.9 to 0.1	399 F g^−1^ at 1A g^−1^	22.5	91% (8000)	2020/^25^
PEDOT-MeOH /SWCNTs	1 M H_2_SO_4_	to 0.5 − 0.8	114.3 mF/cm^−2^ at 5 mV s^−1^	5.3 (μWh cm^−2^)	80% (5000)	2020/^60^
P3HT/SWCNTs	0.1 M LiClO_4_ + CH_3_CN	− 0.2 to 1.0	245.8 F g^−1^ at 0.5 A g^−1^	50.8	80.5% (1000)	This work

*PAN* polyaniline, *PPy* polypyrrole, *TiO*_*2*_ titanium dioxide, *SBS* Poly(styrene-b-butadiene-b-styrene), *[EMIM][NTf2]* 1-Ethyl-3-methylimidazolium bis(trifluoromethylsulfonyl)imide, *PEDOT-PSS* poly(3,4-ethylenedioxythiophene) poly(styrene sulfonate), *Gr* graphene, *PMT* poly(3-methylthiophene), *PD2ET* poly(3-oligo(ethylene oxide))thiophene, *EDOT-MeOH* 3,4-Ethylenedioxythiophene methanol.

## Conclusion

The fabricated supercapacitor electrodes based on P3HT and SWCNTs nanocomposite with different ratios onto a graphite sheet as substrate were carried out. It was found that P3HT/SWCNTs nanocomposite electrodes possess higher specific capacitance than that of each component. The SEM micrographs of the purified SWCNTs have buckypaper structure while the photomicrographs of P3HT/SWCNTs showed that SWCNTs appeared behind and front of the P3HT nanospheres. The specific capacitance of 50% SWCNTs at a current density of 0.5 Ag^−1^ was found to be 245.8 F g^−1^ compared with that of pure P3HT of 160.5 Fg^−1^.

## Experimental section

### Materials

Purified SWCNTs synthetized by SES Research, Houston, TX 77092, USA was received. Poly (3-hexyl-thiophene-2,5-diyl) was purchased from American Dye Source, Inc, USA. Acetonitrile (99.7%) was obtained from Panreac Químican, Spain. Polyvinylidene difluoride (PVDF) powder was obtained from Alfa Aesar, Canada. Ethanol (99.8%), hydrochloric acid (36%) and chloroform (99.4%) were purchased from Sigma-Aldrich Ltd, UK. All chemicals and solvents were of analytical grade and were used without further purification. Graphite sheet with carbon ratio more than 99.5%, density of 1.1 g cm^−3^ and thickness of 0.3 mm was purchased from XRD carbon, China.

### P3HT/SWCNTs electrode fabrication

Graphite sheet was cut to small rectangular shapes with an area of 1 cm^2^. This sheet was treated with 0.1 M HCl in ultrasonic bath for 15 min and washed by ethanol with ultrasonication for 10 min to remove the acid residuals. Subsequently, these sheets were dried at 60 °C for 15 min. The nanocomposite working electrodes were prepared by mixing different amounts of P3HT and SWCNTs as active materials (90%) with 10% PVDF in 1 mL of chloroform, and then subjected to ultrasonication for about 1 h. Next, 20 µL of the resulting homogeneous solution was placed onto the surface of the graphite sheet and dried at 60 °C.

### Characterization techniques

The morphologies of P3HT, SWCNTs and P3HT/SWCNTs composites were characterized by scanning electron microscopy (SEM, JSM-IT200) operated at 20 kV, and transmission electron microscope (HRTEM, JEOL JEM 2100F) at an accelerating voltage of 200 kV. FTIR spectrophotometer (PerkinElmer-Spectrum 2B, USA) was used to identify the structures and function groups of P3HT and P3HT/SWCNTs nanocomposites pressed with KBr. Raman spectrometer (Senterra Bruker, Germany) was used at excitation wavelength of 532 nm.

### Electrochemical measurements

In traditional three-electrode system, the electrochemical performance of P3HT/SWCNTs electrode was tested in 0.1 M LiClO_4_ electrolyte (CH_3_CN as the supporting electrolyte) using OrigaFlex-OGF05 (Origalys, France) electrochemical workstation in which platinum was used as a counter electrode and Ag/AgCl was used as a reference electrode. The potential window for CV tests is from − 0.2 to 1.0 V vs Ag/AgCl at scan rates ranging from 5 to 100 mV s^−1^. At the same potential window GCD was conducted at a current density ranging from 0.5 to 2 Ag^−1^. EIS was measured in the frequency ranging from 10^–2^ to 10^6^ Hz with amplitude of 5 mV at open circuit potential. The related supercapacitor parameters were calculated by the following equations as below^61^:

1 $$C=\frac{I\times\Delta t}{m\times\Delta V},$$

2 $$E=\frac{0.5\times C{\left(\Delta V\right)}^{2}}{3.6},$$

3 $$P=\frac{E\times 3600}{\Delta t},$$

where C, I, and Δt are the specific capacitance (F g^−1^), the charge/discharge current (A), and the discharge time (s). In addition, m is the mass of active material for a single electrode (g), ΔV is the potential window value (V), E is the energy density (Wh kg^−1^) and P is the power density (W kg^−1^). For three electrode system C, E, and P should be dived by 4 to represent an actual device^62^.

## Data Availability

All data included in this study are available upon reasonable request by contact with the corresponding author (A.S.).
